# Photoprotective and Antiaging Effects of a Standardized Red Orange (*Citrus sinensis* (L.) Osbeck) Extract in Asian and Caucasian Subjects: A Randomized, Double-Blind, Controlled Study

**DOI:** 10.3390/nu14112241

**Published:** 2022-05-27

**Authors:** Vincenzo Nobile, Andrea Burioli, Sara Yu, Shi Zhifeng, Enza Cestone, Violetta Insolia, Vincenzo Zaccaria, Giuseppe Antonio Malfa

**Affiliations:** 1 R&D Department, Complife Italia S.r.l., 27028 San Martino Siccomario, PV, Italy; andrea.burioli@complifegroup.com (A.B.); enza.cestone@hotmail.it (E.C.); 2 Clinical Study Department, Complife (Beijing) Testing Technology Co., Ltd., Beizhan North Street N.17, Room 902—Xicheng District, Beijing 100089, China; sarah.yu@complifegroup.cn (S.Y.); giovanni.chen@complifegroup.cn (S.Z.); 3 Active S.r.l., R&D Department, Piano Tavola, 95032 Belpasso, CT, Italy; v.insolia@active-nutra.com; 4 Alma Mater Europea, 6000 Koper, Slovenia; 5 Bionap S.r.l. R&D Department, Piano Tavola, 95032 Belpasso, CT, Italy; 6 Department of Drug and Health Science, University of Catania, Viale A. Doria, 95125 Catania, CT, Italy; g.malfa@unict.it; 7 CERNUT, Research Centre on Nutraceuticals and Health Products, University of Catania, Viale A. Doria, 95125 Catania, CT, Italy

**Keywords:** oral photoprotection, skin aging, oxidative stress, clinical study, red orange extract, food supplement, skin moisturization, skin elasticity

## Abstract

The increase in solar ultraviolet radiation (UVR) that reaches the Earth’s surface should make us reflect on the need to develop new approaches in protecting the skin from UVR exposure. The present study aims to evaluate the photoprotective and antiaging efficacy of a red orange extract (100 mg/day) in both Asian and Caucasian subjects. A randomized, double-blind, controlled study was carried out in 110 Asian and Caucasian subjects. Product efficacy was measured as follows: (1) the photoprotective effect was measured by the minimal erythema dose (MED) assessment; (2) the efficacy in decreasing the UVA+B-induced skin redness was measured by colorimetry; (3) the antioxidant efficacy was measured by the ferric-reducing antioxidant power (FRAP) and the malondialdehyde (MDA) assay; and (4) skin moisturization, skin elasticity, skin radiance, the intensity of melanin staining, transepidermal water loss (TEWL), and wrinkles were measured to assess the antiaging efficacy. The intake of the product for 56 days was effective in improving the skin reaction to UV exposure; in increasing the skin antioxidant capacity as well as in decreasing UVA-induced lipid peroxidation; in increasing the skin moisturization, skin elasticity, and skin radiance; and in decreasing TEWL, the intensity of melanin staining inside dark spots, and wrinkle depth. Our results suggest that the test product is effective in counteracting both the harmful effects of UVR exposure and aging signs.

## 1. Introduction

The long-term changes in cloudiness, ozone, surface reflectivity, and/or aerosols due to anthropogenic activities have increased the solar ultraviolet radiation (UVR) levels around the globe [1,2,3], especially in the mid and high latitudes [4]. Usually, people are exposed to UVR mainly through occupational exposure (i.e., outdoor workers) with recreational UVR exposure (both natural and artificial) increasing over years [5,6,7]. This variation should be correlated to a raised awareness, in the general population, of the harmful effects of UVR, such as erythema, edema, hyperpigmentation, premature aging, and melanoma and non-melanoma skin cancer [6,8]. Excluding the UV-C rays (absorbed by ozone), the UVR reaching the Earth’s surface is predominantly UVA (90–95%) and UVB (5–10%) [9]. The longer wavelength UVA (315–400 nm) penetrates deeply in the skin reaching the dermis, while the shorter wavelength UVB (280–315 nm) are completely absorbed by the epidermis. Each UVR component may exert different effects on the skin. The typical skin reaction to UVR exposure is the induction of inflammation and causes sunburn. This reaction is induced by UVB by the stimulation of a cascade of cytokines and other mediators [10,11,12] that activate apoptotic pathways by keratinocytes when the UV exceeds a threshold damage response [13]. UVB are also involved in the formation of DNA photolesions [14,15]. The reaction to UVA exposure is not visible, and for this reason is more dangerous. UVA exposure is a potent driver of oxidative stress and free radical damage to DNA and other macromolecules [16,17,18,19]. 

The most popular approach in protecting the skin from UVR is the topical application of sunscreens [20]. However, to be effective, the use of topical sunscreen should be part of an integrated photoprotection strategy, including the use of oral photoprotective agents. In recent years, oral supplementation with a wide variety of polyphenols, carotenoids, or other phytochemicals has been reported to possess substantial skin photoprotective effects [21,22]. However, despite the extensive range of food supplements with the most disparate range of health claims, a few of these products claim a positive effect in improving skin appearance, including skin photoprotection. To make matters worse, most of these products rely on claims supported by scarce scientific studies [21,23].

In the current study, we are interested in investigating the efficacy of a standardized Red Orange Complex extract (ROC^TM^, Bionap Srl, Piano Tavola Belpasso, Italy) containing anthocyanins, hydroxycinnamic acids, flavanones, and ascorbic acid on humans. Previous in vitro studies demonstrated the antioxidant [24], UVB protection [25], and anti-inflammatory [26] efficacy of the extract in a cell line of human keratinocytes, while in vivo studies on humans demonstrated its efficacy in decreasing oxidative stress in subjects exposed to air pollution [27] and its photoprotective efficacy on UVB-induced erythema [28] and photoaging [29]. The present study aims to explore more in detail the photoprotective and the antiaging efficacy of the extract in both Asian and Caucasian populations. 

## 2. Materials and Methods

### 2.1. Description of Study Design

This was a multicenter, stratified (Asian and Caucasian ethnicity and male and female subjects, with imbalanced randomization [1.5:1]), randomized, double-blind, placebo-controlled study conducted in Beijing (China) and Milan (Italy). 

All the study procedures were carried out according to the World Medical Association’s (WMA) Helsinki Declaration and its amendments (Ethical Principles for Medical research Involving Human Subjects, adopted by the 18th WMA General Assembly Helsinki, Finland, June 1964, and amendments). The study protocol and the informed consent form were approved by the “Independent Ethical Committee for Non-Pharmacological Clinical trials” (ref. 2020/07). The trial is registered at ISRCTN registry, number ISRCTN89244753, https://doi.org/10.1186/ISRCTN89244753.

All the subjects signed the informed consent and consent release forms for the publication of photographs before any study-related procedures took place.

### 2.2. Eligibility Criteria for Participants

Eligible participants were all healthy adults aged between 35 and 55 years (extremes included) with mild to moderate skin aging signs (grade 2–4; Skin Aging Atlas by Bazin R. [30,31]) who met the following inclusion criteria: female and male sex (between 30–50% male and 50–70% female sex), skin phototype (Fitzpatrick classification) in the range from I to V (I–III for Caucasian and III–V for Asian subjects), and dark spots. Exclusion criteria were chosen to recruit a healthy study population. A list of the exclusion criteria can be found in Supplementary Table S1.

### 2.3. Settings and Locations

The study took place at Complife Italia Srl (San Martino Siccomario, Italy) and at Complife (Beijing) testing technology Co., Ltd. (Beizhan North Street N.17, Room 902—Xicheng District, Beijing, China). Complife is an independent testing international laboratory specialized in the in vitro and in vivo safety and efficacy assessment of cosmetics, food supplements, and medical devices.

### 2.4. Intervention

The participants were randomly assigned, by a computer-generated randomization sequence, to receive the active and the placebo product. The active product was a food supplement containing a standardized Red Orange Complex extract (ROC^TM^, Bionap Srl, Piano Tavola Belpasso, Italy) obtained from 3 different pigmented, red, and Sicilian orange (*Citrus sinensis*) varieties (Moro, Tarocco, and Sanguinello). In total, this complex contains (*w*/*w*) the following antioxidants: 2.8–3.2% anthocyanins (cyanidin-3-glucoside), 1.8–2.2% hydroxycinnamic acids, 8.5–9.5% flavanones (hesperidin and narirutin), and 5.5–6.5% ascorbic acid. Each capsule of the active food supplement contained 100 mg Red Orange Complex H, 200 mg maltodextrin, 108 mg capsule jelly size 0, and 2 mg titanium dioxide. The placebo product contained (per capsule) 300 mg maltodextrin, 108 mg capsule jelly size 0, and 2 mg titanium dioxide. The posology for both the active and the placebo products was 1 capsule a day after breakfast.

### 2.5. Primary and Secondary Objectives and Outcome Measures

The primary objective of the study was the assessment of the efficacy of the product in decreasing the UV-induced erythema reaction before and after 8 weeks of the product intake period. The primary outcome measures were the minimal erythema dose measurement and the instrumental measurement of skin redness.

The secondary objective was the assessment of the antiaging efficacy of the product. The secondary outcome measures were: skin moisturization (measured by means of Corneometer^®^ CM 825, Courage + Khazaka electronic GmbH, Cologne, Germany), skin elasticity (measured by means of Cutometer^®^ MPA 580, Courage + Khazaka electronic GmbH, Cologne, Germany), transepidermal water loss (measured by means of Tewameter^®^ TM 300, Courage + Khazaka electronic GmbH, Cologne, Germany), total skin antioxidant capacity (FRAP assay), skin lipoperoxide content (MDA assay), intensity of melanin staining (colorimetric method), skin radiance (colorimetric method), and wrinkle depth, length, area and wrinkle count (skin profilometry by structured light 3D scanner and clinical scoring).

**Figure 1:**
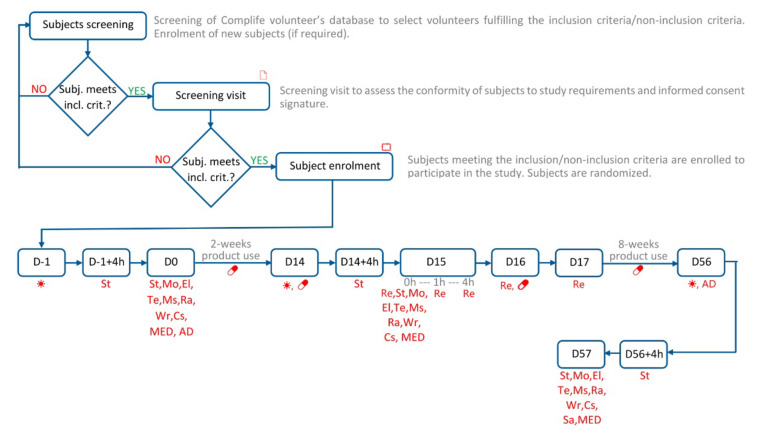
Study flow and schedule of assessment chart. Legend. 🗋, Informed consent signature; ⮔, Randomization; ☀, UVA+B and UVA exposure; 💊 product intake; St, Skin stripping (collection of skin samples for FRAP and MDA); Mo, Skin moisturization; El, Skin elasticity; Te, Transepidermal water loss; Ms, Intensity of melanin staining; Ra, Skin radiance; Wr, Wrinkle measurement; Cs, Clinical scoring; Sa, Self-assessment questionnaire; MED, Minimal Erythema Dose; Re, Skin redness; AD, Alimentary Diary (from D0 to D56).

The study flow and the schedule of assessment chart are shown in Figure 1. Information on the measurement’s sites for each parameter can be found in Supplementary Figure S1.

#### 2.5.1. UV Exposure

A selected area on the back (for MED and UV-induced redness assessment) or on the legs (for antioxidant effect assessment) were exposed to UV radiation. 

The source of both UVA+B and UVA radiation was a Multiport 601–300 W Solar simulator (Solar^®^ Light Co., Inc., Philadelphia, PA, USA) compliant with ISO 24444:2010 standard and with the Japan Cosmetic Industry Association (JCIA) measurement standard for UVA protection/ISO 24442:2011 standard requirements, respectively. The UVB and the UVA dose were adjusted with a model PMA 2100 radiometer (Solar^®^ Light Co., Inc., Philadelphia, PA, USA) equipped with a PMA 2103 LLG SUV detector (Solar^®^ Light Co., Inc., Philadelphia, PA, USA) or with a PMA 2113 LLG UVA detector (Solar^®^ Light Co., Inc., Philadelphia, PA, USA), respectively. Both the solar simulator and the radiometers were calibrated externally (solar simulator spectral characteristics can be found in Supplementary Figure S2).

#### 2.5.2. Minimal Erythema Dose (MED) Measurement and UV-Induced Skin Redness

The minimal erythemal dose (MED) is the lowest dose of ultraviolet radiation (UVR) that produces the first perceptible unambiguous erythema with defined borders appearing over most of the field of UV exposure, 20 ± 4 h after UV exposure. MED was performed by applying a series of UV exposures, using a model 601–300 W solar simulator (Solar Light Co., Inc., Philadelphia, PA, USA) according to the skin phototype of the subject [32]. The back (within the region between the scapula line and the waist) was chosen as the anatomical region for the test area.

#### 2.5.3. Antioxidant Properties Measurement

Both the total antioxidant capacity and the skin lipoperoxide content were measured on skin strippings taken using Corneofix^®^ foils (Courage + Khazaka electronic GmbH, Cologne, Germany). Skin stripping numbers 2 and 3 were collected for FRAP assay, while skin stripping numbers 10 and 11 were collected for the MDA assay. Skin strippings were stored at −80 °C after their collection for the FRAP and MDA assays.

The total skin antioxidant capacity was measured by means of the ferric-reducing antioxidant parameter (FRAP). FRAP is a direct measure of the total reductive power of a biological matrix and an indirect index of the capability of the considered system to resist oxidative damage. FRAP uses the antioxidants in the biological system as a reductive agent in a colorimetric method based on redox reactions [33,34]. The reduction at an acid pH of the complex TPTZ–Fe(III) in the ferrous form (Fe(II)) is characterized by an intense blue color. The reaction was monitored by measuring the solution absorbance at 595 nm. The recorded absorbance was compared to a Fe(II) standard curve of known values. The results were directly proportional to the total reductive power of the antioxidant in the reaction mix.

Lipoperoxides were measured by means of a Malonyldialdehyde assay (MDA). Malonyldialdehyde (MDA) and 4-hydroxynonenal are the two main products of lipid peroxidation. Their concentration in a biological system is a good index of its lipoperoxide damage. The lipoperoxide levels were assayed using the method developed by Erdelmeier and collaborators [35]: the assay is based on the capability of the chromogen, N methyl 2 phenylindole (NMPI), to react with MDA at 45 °C and an acid pH to produce a stable chromophore that has an absorption peak at 586 nm. The lipoperoxide levels were measured after the induction of unstable hydroperoxide decomposition, produced in the oxidative processes by means of a pro-oxidant agent (CuSO_4_ 500 mM). Lipoperoxides were measured before and after 4 and 24 h of UV exposure. 

#### 2.5.4. Skin Bioengineering Techniques

- The measurement of skin moisturization was based on the Corneometer^®^ method. The corneometer^®^ method is based on the dielectric constant of water. The probe shows changes of capacitance according to the moisture content of the skin. An electric scatter field penetrates the very first layers of the skin (10–20 µm) and determines the dielectricity. The used device was the Corneometer^®^ CM 825 (Courage + Khazaka, electronic GmbH, Cologne, Germany).
- The measurement of skin elasticity was based on the suction method using a negative pressure mechanically deforming the skin (Cutometer^®^ method). A negative pressure (450 mbar) is created in the device and the skin is drawn into the aperture of the probe for 2 s and after a defined time (2 s) it is released again. Inside the probe, the penetration depth is determined by a non-contact optical measuring system. The optical measuring system consists of a light source and a light receptor, as well as two prisms facing each other, which project the light from the transmitter to the receptor. The light intensity varies due to the penetration depth of the skin. The resistance of the skin to the negative pressure and its ability to return into its original position are displayed as curves (penetration depth in mm/time) in real time during the measurement. The used device was the Cutometer^®^ MPA 580 (Courage + Khazaka, electronic GmbH, Cologne, Germany). Skin elasticity was measured in the cheek. R0 (skin distensibility), R2 (overall skin elasticity), R5 (net elasticity), and R9 (skin tiring effect) parameters were measured. More information on skin elasticity parameters can be found in Supplementary Figure S3.
- Transepidermal water loss (TEWL) was measured using a Tewameter^®^ TM 300 (Courage + Khazaka, electronic GmbH, Cologne, Germany). The measurement is based on the diffusion law. The diffusion flow dm/dt indicates the mass of water, which is transported per cm² in a specific period. The resulting density gradient is measured indirectly by two pairs of sensors (temperature and relative humidity) and is analyzed by a microprocessor. The measuring head of the probe was a narrow hollow cylinder (10 mm diameter and 20 mm height) to minimize the influence of air turbulence inside the probe.
- The intensity of melanin staining inside dark spots was assessed by means of the Individual Typology Angle (ITA°) parameter [3]. ITA° is calculated starting from L* and b* values measured in the CIELab space (1976) using a spectrophotometer/colorimeter CM-700D (Konica-Minolta, Japan).
- The skin radiance (or skin brightness) is the ability of the skin to reflect the light and was measured using the gloss parameter taken using a spectrophotometer/colorimeter CM-700D (Konica-Minolta, Japan). The instrument emits diffused light that reaches the skin through an opening located at the extreme of the lighting sphere. A sensor located at 8° compared to the vertical axis of the opening detects then the reflected light and calculates a parameter known as “gloss”. The gloss value is used in the management of the brilliance of the color.
- Wrinkle depth, length, area, and wrinkle count in the “crow’s feet” area. were measured using a real 3D microtopography imaging system (Primos^CR^ SF, Canfield Scientific, NJ, USA). Skin surface was reconstructed using an algorithm to generate 3D images. Subject repositioning was ensured by a repositioning device (Canfield Scientific, NJ, USA), while before/after image matching was ensured by an overlapping feature of the image analysis software.
- The dermatologists evaluated the skin appearance (wrinkle appearance and dark spot decrease) using a clinical scoring system (−3 greatly worsened, −2 moderately worsened, −1 slightly worsened, 0 no change, +1 slightly improved, +2 moderately improved, +3 greatly improved).

#### 2.5.5. Anthropometric Measurements

Height, weight, BMI, waist, and hip circumference were measured before and after product use.

### 2.6. Randomization and Masking

Half of the participants were randomized to receive the test product and half of the participants were randomized to receive the placebo product. A restricted randomization list was created using PASS 11 (vers. 11.0.10; PASS, LLC. Kaysville, UT, USA) statistical software running on Windows Server 2008 R2 Standard SP1 64-bit Edition (Microsoft, Redmond, WA, USA) by a biostatistician and stored in a safe place. The randomization sequence was stratified using “Efron’s biased coin” algorithm with a 1:1 allocation ratio. The allocation sequence was concealed from the study director in sequentially numbered, opaque, and sealed envelopes, reporting the unblinded treatment allocation (based on subject entry number in the study). The A4 sheet reporting the unblinded treatment was folded to render the envelope impermeable to intense light. A masked allocation sequence was prepared for the staff delivering the intervention based on the subject entry number in the study. An independent technician dispensed either active or placebo products according to the masked allocation sequence. The study adhered to established procedures to maintain separation between the investigator and the collaborators and the staff that delivered the intervention. The investigator and the collaborators who obtained outcome measurements were not informed on the (masked) product group assignment. The staff who delivered the intervention did not take outcome measurements. Subjects, investigator, and collaborators were kept masked to product assignment.

### 2.7. Statistical Analysis

Statistical analysis was performed using NCSS 10 (version 10.0.7 for Windows; NCSS, Kaysville, UT, USA) running on Windows Server 2008 R2 Standard SP1 64-bit edition (Microsoft, Redmond, WA, USA). Parametric data were submitted to *t*-test, while non-parametric data were submitted to Wilcoxon (intragroup analysis) or Mann–Whitney tests (intergroup analysis). Data normality was checked by Shapiro–Wilk W test. A *p* < 0.05 was considered statistically significant. Statistical analysis output was reported as follows: * *p* < 0.05, ** *p* < 0.01, and *** *p* < 0.001.

## 3. Results

### 3.1. Participants and Product Tolerability

**Figure 2:**
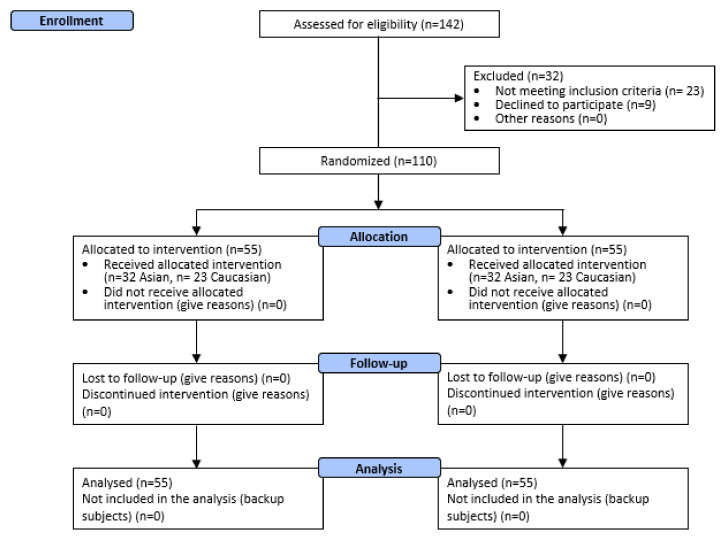
Participant flow diagram.

A total of 110 male and female subjects were successfully randomized. Fifty-five (*n* = 55) subjects were allocated to each treatment arm (Figure 2.). The population was Asian (*n* = 32 per each treatment arm) and Caucasian (*n* = 23 per each treatment arm). Asian subjects were enrolled in China, in the Beijing geographical area, while Caucasian subjects were enrolled in Italy in the geographical area between Pavia and Milano (Lombardia region). Demographic and baseline characteristics (Supplementary Table S2) were similar across treatment arms, indicating an unbiased randomization and the absence of covariates. No dropouts were recorded. All subjects were included in the efficacy and safety analysis dataset. All the tested products were well tolerated. No adverse reactions occurred during the study period.

### 3.2. Primary Endpoints: MED and UV-Induced Erythema

**Figure 3:**
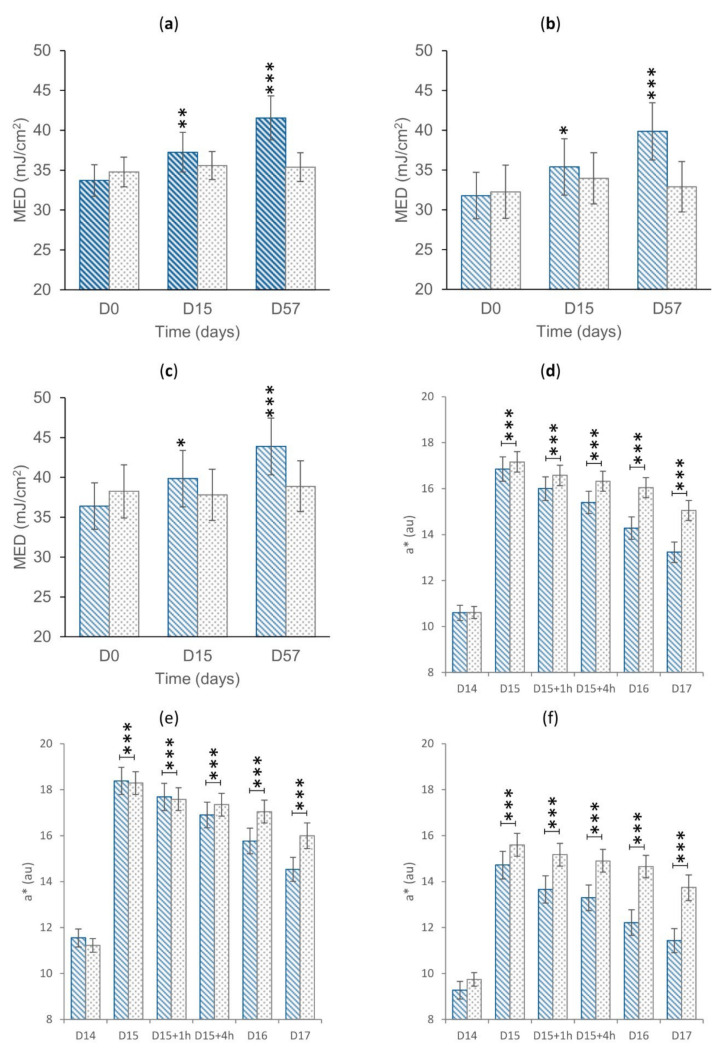
(**a**) MED in the overall treatment arm. (**b**) MED in the Asian treatment arm. (**c**) MED in the Caucasian treatment arm. Data are average (±standard error). (**d**) Skin redness in the overall treatment arm. (**e**) Skin redness in the Asian treatment arm. (**f**) Skin redness in the Caucasian treatment arm. The intragroup (graphs **a**–**c**) and the intergroup (graphs **d**–**f**) statistical analyses are reported above the bar as follows: * *p* < 0.05, ** *p* < 0.01, and *** *p* < 0.001. Legend. au, arbitrary units. D0, baseline. D15, follow-up visit after 14 days of product use; D15+1h, 1 h after the product intake at D15; D15+4h, 4 h after the product intake at D15; D16, 1 day after the D15 product intake; D17, 2 days after the D15 product intake; D57, follow-up visit after 56 days of product use. 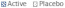.

The primary endpoints related to efficacy were measured before and after UVA+B exposure using a solar simulator. Data are reported in Figure 3. 

The variation of MED was statistically significant (*p* < 0.05) vs. D0 for all the subgroups of the active treatment arm (overall: +10.0 and +22.2% after 14 and 56 days of product use, respectively; Asian: +10.0 and +22.5% after 14 and 56 days of product use, respectively; Caucasian: +9.4 and +21.7% after 14 and 56 days of product use), while not being statistically significant for the placebo treatment arm (except for the Asian subgroup at D15). After 56 days of product use, the variation of MED was statistically higher for the active arm when compared to the placebo arm for all the subgroups (overall *p* < 0.001, Caucasian *p* < 0.01, and Asian *p* < 0.001). 

The variation of the UV-induced skin redness was statistically significant (*p* < 0.05) vs. D0 for all the subgroups of the active and placebo treatment arms (overall, Asian, Caucasian). At D15+1h in the overall (−4.9% vs. placebo) and in the Caucasian (−7.4% vs. placebo) treatment arm, the variation of the UV-induced skin redness was statistically lower for the active arm when compared to the placebo arm (overall *p* < 0.01 and Caucasian *p* < 0.05). At D15+4h, the variation of the UV-induced skin redness was statistically lower for the active arm when compared to the placebo arm for all the subgroups (overall: −8.1%, *p* < 0.001; Asian: −7.7%, *p* < 0.05; Caucasian: −8.8% *p* < 0.05). At D16 and D17, the variation of the UV-induced skin redness was statistically lower for the active arm when compared to the placebo arm for all the subgroups (overall: −16.5% and −16.9% at D16 and D17, respectively, *p* < 0.001; Caucasian: −18.3% and −17.8% at D16 and D17, respectively, *p* < 0.001; Asian −15.1% and −16.1% at D16 and D17, respectively, *p* < 0.001). The differences between the active and treatment arms indicated an effect of the product in improving the skin reddening reaction to UV radiation exposure.

These data support the effect of the product in improving the skin behavior to sun exposure. In particular, regular product use can have a positive effect in increasing the minimal erythema dose and in accelerating the resolution of the UV-induced skin redness.

### 3.3. Secondary Endpoints

#### 3.3.1. Antioxidant Efficacy

The antioxidant efficacy was measured both as the increase in the skin total antioxidant capacity by the FRAP assay and as the decrease in the baseline and UVA-stimulated lipid peroxidation by MDA assay. FRAP and basal MDA data are reported in Table 1. UVA-stimulated MDA data are reported in Supplementary Table S3.

The total antioxidant capacity of the skin was statistically significantly increased (overall: +8.6 and +22.3% after 14 and 56 days of product use, respectively; Asian: +8.5 and +22.0% after 14 and 56 days of product use, respectively; Caucasian: +8.8 and +22.6% after 14 and 56 days of product use, respectively) in all the active-treated groups (overall, Asian, and Caucasian), while not being statistically significant in the placebo-treated groups. Differences in the total antioxidant capacity variation (vs. D0) were statistically significant between active and placebo (D15 overall *p* < 0.001, D15 Caucasian *p* < 0.01, and D15 Asian *p* < 0.01; D57 overall *p* < 0.001, D57 Caucasian *p* < 0.001, and D57 Asian *p* < 0.001) for the overall, Asian, and Caucasian groups. 

The basal level of lipid peroxides (MDA) was statistically significantly decreased (overall: −1.5 and −8.2% after 14 and 56 days of product use, respectively; Asian: −7.7% after 56 days of product use; Caucasian: −1.9 and −8.9% after 14 and 56 days of product use, respectively) in all the active-treated groups, while not being statistically significant in the placebo-treated groups. Differences in the total antioxidant capacity variation (vs. D0) were statistically significant between active and placebo (D14 overall *p* < 0.01, D14 Caucasian *p* < 0.05; D56 overall *p* < 0.001, D56 Caucasian *p* < 0.001, and D56 Asian *p* < 0.001) for the overall, Asian, and Caucasian groups.

The decrease in UVA-stimulated skin lipoperoxide content (Supplementary Table S3) was statistically significant when compared to the placebo groups, starting at D56 + 4h for the Asian subgroup (*p* < 0.05) and for the overall group (*p* < 0.01) and at D57 Caucasian group (*p* < 0.05). In conclusion, the product was effective in decreasing the skin lipoperoxide content variation after UVA exposure starting at D56 days of product use in the overall group and in the Asian subgroup, while at D57 in the Caucasian subgroup.

#### 3.3.2. Skin Antiaging Effect

The antiaging effect of the product was measured by means of non-invasive skin bioengineering techniques. The following parameters were measured: skin moisturization, skin elasticity, skin radiance, the intensity of melanin staining of dark spots, transepidermal water loss (TEWL), and wrinkles (depth, length, area, and wrinkle count). Data are reported in Table 2 and in Supplementary Table S4.

Skin moisturization was statistically significantly increased (overall: +5.3 and +12.3% after 14 and 56 days of product use, respectively; Asian: +4.8 and +11.0% after 14 and 56 days of product use, respectively; Caucasian: +14.2% after 56 days of product use) in all the active-treated groups, while not being statistically significant in the placebo-treated groups, except for a random statistically significant variation at D15 in the overall group. The increase in skin moisturization started at D15 for the overall and the Asian group and at D57 for the Caucasian group. 

The skin radiance (or brightness) was statistically significantly increased (overall: +9.2 and +18.5% after 14 and 56 days of product use, respectively; Asian: +8.0 and +18.2% after 14 and 56 days of product use, respectively; Caucasian: +10.9 and +18.8% after 14 and 56 days of product use, respectively) in all the active-treated groups, while not being statistically significant in the placebo-treated groups. 

The individual typology angle (ITA°) inside dark spots was statistically significantly increased (overall: +14.8 and +32.9% after 14 and 56 days of product use, respectively; Asian: +15.3 and +33.2% after 14 and 56 days of product use, respectively; Caucasian: +14.1 and +32.4% after 14 and 56 days of product use, respectively) in all the active-treated groups, while being statistically significant in the placebo-treated groups only at D57. The increase in ITA° indicates a decrease in melanin staining inside dark spots. This instrumentally measured data were also seen by the dermatologists in most of the subjects (overall: 41.8 and 71.7% after 14 and 56 days of product use, respectively; Asian: 37.5 and 71.9% after 14 and 56 days of product use, respectively; Caucasian: 47.8 and 73.9% after 14 and 56 days of product use, respectively) participating in the study. In the placebo-treated group, a mild decrease in dark spot appearance was reported in about 30% of the subjects both at D15 and D57. 

The transepidermal water loss (TEWL) was statistically significantly decreased (overall: −2.7 and −14.5% after 14 and 56 days of product use, respectively; Asian: −4.0 and −14.3% after 14 and 56 days of product use, respectively; Caucasian: −14.9% after 56 days of product use, respectively) in all the active-treated groups, while not being statistically significant in the placebo-treated groups. The decrease in TEWL started at D15 for the Overall and the Asian groups and at D57 for the Caucasian group.

**Figure 4:**
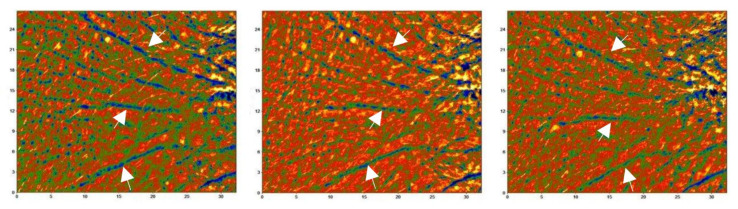
Primos CR pictures before and after product use. The picture shows the product effect in decreasing the mean deepest wrinkle in one of the subjects showing the best effect. A decrease in wrinkle depth (arrows) can be seen as a decrease in the darker colors in the LUT below each image. The image size is 27 (h) × 32 (w) mm. Legend. 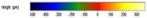 (from −500 µm [black] to 350 µm [yellow]).

The change in the mean deepest wrinkle (Figure 4) was statistically significant at D57 (overall: −6.9%; Asian: −7.5%; Caucasian: −6.2%) in all the active-treated groups, while not being statistically significant in the placebo-treated groups (except at D15 in the Asian treatment group, with minimal worsening of wrinkle depth). All the other parameters related to wrinkledness (length, area, and wrinkle count) did not statistically significantly change. This instrumentally measured data were also seen by the dermatologists in most of the subjects (overall: 5 and 40.0% after 14 and 56 days of product use, respectively; Asian: 6.3 and 40.6% after 14 and 56 days of product use, respectively; Caucasian: 4.3 and 39.1% after 14 and 56 days of product use, respectively) participating in the study. In the placebo-treated group, a mild decrease in wrinkle appearance was reported in less than 10% of the subjects both at D15 and D57. 

The anthropometric parameters measured in the study (Supplementary Table S5) were not statistically significant between the active and placebo treatment arms for all the groups. This indicates that the product does not have any slimming or any other actions in weight management. 

The product effect on skin elasticity was measured by means of four skin elasticity parameters, as follows: skin distensibility (R0), overall skin elasticity (R2), net elasticity (R5), and skin tiring effect (R9). Data are reported in Table 3. The skin distensibility parameter (R0) was statistically significantly decreased (overall: −8.2% and −15.6% after 14 and 56 days of product use, respectively; Asian: −9.3 and −15.2% after 14 and 56 days of product use, respectively; Caucasian: −6.5 and −16.0% after 14 and 56 days of product use, respectively) in all the active-treated groups, while not being statistically significant in the placebo-treated groups (except at D15 in the overall group, with minimal worsening skin distensibility). The skin overall elasticity parameter (R2) was statistically significantly increased (overall: +5.9 and +13.9% after 14 and 56 days of product use, respectively; Asian: +6.2 and +15.5% after 14 and 56 days of product use, respectively; Caucasian: +5.5 and +11.6% after 14 and 56 days of product use, respectively) in all the active-treated groups, while not being statistically significant in the placebo-treated groups.

The skin net elasticity parameter (R5) was statistically significantly increased (overall: +16.8 and +33.4% after 14 and 56 days of product use, respectively; Asian: +15.9 and +34.3 after 14 and 56 days of product use, respectively; Caucasian: +17.1 and +31.0% after 14 and 56 days of product use, respectively) in all the active-treated groups, while not being statistically significant in the placebo-treated groups. 

The skin tiring effect parameter (R9) was statistically significantly decreased (overall: −12.1 and −25.4% after 14 and 56 days of product use, respectively; Asian: −12.1 and −25.8% after 14 and 56 days of product use, respectively; Caucasian: −11.9 and −23.8% after 14 and 56 days of product use, respectively) in all the active-treated groups, while not being statistically significant in the placebo-treated groups (except at D15 in the Asian treatment group, with mild worsening of skin tiring effects).

## 4. Discussion

Skin health is not only undermined by internal general aging processes, but also by environmental stressors, changing not only its appearance but also its physiological functions [36]. Among others, UVR exposure accelerates the rate of skin degeneration over skin regeneration. This effect is variable according to global location and skin type, even if the link between chronic sun exposure and harmful clinical consequences, such as photoaging and melanoma and non-melanoma skin cancer, is now indisputable [37,38]. 

A global approach to aging and photoprotection is then of primary importance to maintain skin health. In this context, the use of food supplements with both antiaging and photoprotective efficacy has increased in recent years [38,39]. Eating well could be the best way to defend our skin. This is the new awareness of the role of nutrition in skin health and specific dietary components have emerged as an effective alternative strategy to prevent and alleviate the symptoms of photoaging. However, despite their widespread use, most of these products rely on scarce scientific studies [21,23], even if, recently, the number of studies on skin-functioning food supplements has increased [40,41]. Therefore, in this study, we investigated the efficacy of a standardized red orange (*Citrus sinensis* (L.) Osbeck) extract (Red Orange Complex) obtained from the juice of three pigmented (Moro, Tarocco, and Sanguinello) varieties of Sicilian blood orange grown exclusively in a particular area surrounding Europe’s most active volcano, Mt. Etna (Catania, CT, Italy). This extract contains anthocyanins (cyanidin-3-glucoside), hydroxycinnamic acids, flavanones (hesperidin, narirutin), and ascorbic acid. 

The beneficial role of the test product on UV-induced skin deterioration has been reported both in vitro and in vivo [24,25,26,27,28,29]. In this study, we integrated this information in a single multiethnic study to better understand the role of the test product in skin photoprotection. The administration of the test product decreased the susceptibility of the skin to sun exposure (MED increase) starting from 14 days of product use. Under real-life conditions, this effect is related to an increase in the UVR dose needed to produce skin reddening. This effect was also confirmed by the acceleration of the UV-induced skin redness. 

This study confirmed also the general well-known antioxidant efficacy of *Citrus sinensis* to the skin by the skin stripping technique. The total skin antioxidant capacity (both enzymatic and non-enzymatic) of the stratum corneum was increased starting from 14 days of product intake. The increase in the total antioxidant capacity was confirmed also by the decrease in both the basal and the UVA-stimulated by-products of lipid peroxidation. This confirms the antioxidant network of the stratum corneum and its biomarker role of environmentally induced oxidation [34,42]. 

The antiaging efficacy of the test product was demonstrated by the increase in skin moisturization, skin elasticity, and skin radiance and by the decrease in TEWL, the intensity of melanin staining inside dark spots, and wrinkle depth. Similar effects were reported by Tamaru and colleagues [43] in mice. In this study, the authors reported the positive effect of the oral administration of immature *Citrus unshiu* powder on improving the UVB-induced loss of skin hydration, an increase in transepidermal water loss, and the overgrowth of epidermal cells, while suppressing epidermal cell mortality and basement membrane destruction in hairless mice. 

The improvement in skin elasticity parameters demonstrated (indirectly) the effect of the test product on the extracellular matrix components, as follows: R0 decrease correlates with the stretching of both collagen and elastic fibers and is inversely proportional to their thickness and rigidity [44,45]; R2 increase is related to the function of the elastic fibers of the skin [46]; R5 increase is an index of reduced skin aging [44]; and R9 decrease represents the resilience of the skin. This positive effect could be also related to the key role of ascorbic acid in collagen biosynthesis by the promotion of the expression of collagen genes and by its stabilizing role in the collagen molecule tertiary structure [47]. The product’s effect on improving skin elasticity could be correlated with its protective effect on extracellular matrix remodeling, as previously described by Tomasello et al. [29]. According to the study findings, in a human foreskin fibroblast (HFF-1) cell culture model, the *C. sinensis* extract was effective in: (a) restoring the UVB-induced downregulation of the transcriptional and translational levels of type I collagen and elastin, and (b) in reducing the UVB-stimulated matrix metalloproteinase (MMP-1 and MMP-9) mRNA expression and protein levels, in a concentration-dependent manner. The reason for the significant improvements in skin parameters shown in this study might be related to an improvement in the metabolic reactions of the skin due to its strong antioxidant properties [39,43,48]. 

Interestingly, a beneficial effect of orange extract supplementation was demonstrated on Caenorhabditis elegans’s lifespan [49].

## 5. Conclusions

The intake of 100 mg/day of the standardized Red Orange Complex extract containing anthocyanins, hydroxycinnamic acids, flavanones, and ascorbic acid was effective in counteracting both the harmful effects of UVR exposure and aging signs. To the best of our knowledge, this is the first study reporting, in the meantime, the photoprotective and antiaging efficacy of a *Citrus sinensis* extract in Asian and Caucasian subjects.

## Figures and Tables

**Table 1:** FRAP and basal MDA results. The intragroup (vs. baseline) statistical analysis is reported near the raw data, while the intergroup (active vs. placebo) statistical analysis is reported near the % variation (Δ%). The statistical analysis is reported as follows: * *p* < 0.05, ** *p* < 0.01, and *** *p* < 0.001. Legend. D0, baseline (first day of study, exposure of skin to UV); D−1, day before the basal visit (before UV exposure); D14/15, follow-up visit after 14 days of product use; D56/57, follow-up visit after 56 days of product use. O, Overall (Asian and Caucasian subjects); A, Asian; C, Caucasian.

		Active					Placebo				
		D0	D15	Δ%	D57	Δ%	D0	D15	Δ%	D57	Δ%
**FRAP** (μM Fe^2+^)	**O**	329.0 ± 8.4	356.7 ± 9.5 ***	+8.6% ***	402.3 ± 12.6 ***	+22.3% ***	333.8 ± 9.3	335.1 ± 10.5	+0.4%	339.3 ± 10.2	+2.0%
	**A**	321.2 ± 11.3	347.1 ± 11.9 ***	+8.5% **	391.9 ± 17.0 ***	+22.0% ***	331.3 ± 10.5	331.4 ± 12.1	+0.1%	337.7 ± 11.4	+2.2%
	**C**	339.9 ± 12.6	370.0 ± 15.4 ***	+8.8% ***	416.8 ± 18.8 ***	+22.6% ***	337.2 ± 17.0	340.4 ± 18.9	+0.8%	341.5 ± 18.9	+1.7%
		**D−1**	**D14**		**D56**		**D−1**	**D14**		**D56**	
**MDA** (μM MDA)	**O**	16.8 ± 0.6	16.5 ± 0.6	−1.5% **	15.4 ± 0.6 ***	−8.2% ***	16.9 ± 0.6	17.4 ± 0.6	+3.7%	17.3 ± 0.6	+3.1%
	**A**	16.4 ± 0.8	16.1 ± 0.8	−1.2%	15.0 ± 0.8 ***	−7.7% ***	17.3 ± 0.8	17.6 ± 0.8	+2.4%	17.7 ± 0.8	+3.2%
	**C**	17.5 ± 0.9	17.1 ± 0.8	−1.9% *	16.0 ± 0.8 ***	−8.9% ***	16.4 ± 0.8	17.1 ± 0.8	+5.5%	16.7 ± 0.8	+2.9%

**Table 2:** Secondary endpoints results. The intragroup (vs. baseline) statistical analysis is reported near the raw data, while the intergroup (active vs. placebo) statistical analysis is reported near the % variation (Δ%). The statistical analysis is reported as follows: * *p* < 0.05, ** *p* < 0.01, and *** *p* < 0.005. Legend. D0, baseline; D14, follow-up visit after 15 days of product use; D57, follow-up visit after 56 days of product use. O, Overall (Asian and Caucasian subjects); A, Asian; C, Caucasian.

		Active					Placebo				
		D0	D15	Δ%	D57	Δ%	D0	D15	Δ%	D57	Δ%
**Moist. (cu)**	**O**	53.7 ± 2.2	55.9 ± 2.2 ***	+5.3% **	59.3 ± 2.2 ***	+12.3% ***	55.1 ± 2.2	51.6 ± 2.0 *	−3.6%	53.2 ± 2.0	−1.9%
	**A**	61.7 ± 2.1	64.3 ± 2.0 ***	+4.8% *	67.9 ± 2.0 ***	+11.0% ***	62.6 ± 2.3	58.3 ± 2.1	−4.1%	60.8 ± 1.9	−1.8%
	**C**	42.6 ± 3.3	44.3 ± 3.1	+5.9%	47.3 ± 3.0 ***	+14.2% ***	44.7 ± 3.0	42.2 ± 2.8	−2.8%	42.8 ± 2.9	−2.0%
**Rad. (au)**	**O**	10.9 ± 0.5	11.5 ± 0.5	+9.2%	12.5 ± 0.6 ***	+18.5% ***	11.6 ± 0.5	11.6 ± 0.5	+1.8%	11.7 ± 0.5	+2.5%
	**A**	10.5 ± 0.7	10.8 ± 0.5	+8.0%	12.1 ± 0.7 ***	+18.2% ***	10.6 ± 0.6	10.6 ± 0.7	+1.3%	10.9 ± 0.7	+2.4%
	**C**	11.4 ± 0.9	12.4 ± 1.0 *	+10.9%	13.2 ± 1.0 ***	+18.8% **	13.0 ± 0.9	12.9 ± 0.7	+2.5%	12.9 ± 0.7	+2.4%
**ITA°**	**O**	23.0 ± 1.3	25.7 ± 1.4 ***	+14.8%	29.3 ± 1.5 ***	+32.9% ***	23.5 ± 1.1	24.3 ± 1.2	+6.8%	25.7 ± 1.2 **	+12.5%
	**A**	23.4 ± 1.8	26.1 ± 2.0 ***	+15.3%	29.7 ± 2.1 ***	+33.2% **	24.1 ± 1.7	25.0 ± 1.8	+7.4%	26.4 ± 1.9 *	+13.2%
	**C**	22.4 ± 1.7	25.2 ± 2.0	+14.1% ***	28.9 ± 2.1 ***	+32.4% **	22.8 ± 1.4	23.2 ± 1.3	+5.8%	24.7 ± 1.4 *	+11.5%
**TEWL (g/h/m^2^)**	**O**	12.7 ± 0.5	12.2 ± 0.4	−2.7% **	10.8 ± 0.4 ***	−14.5% **	12.2 ± 0.5	12.9 ± 0.5	+8.0%	12.6 ± 0.4	+4.8%
	**A**	13.3 ± 0.7	12.5 ± 0.5	−4.0% *	11.2 ± 0.5 ***	−14.3% ***	13.2 ± 0.7	13.7 ± 0.6	+6.4%	13.4 ± 0.6	+3.5%
	**C**	11.9 ± 0.6	11.7 ± 0.6	−1.0%	10.1 ± 0.6 ***	−14.9% ***	10.9 ± 0.5	11.9 ± 0.7	+10.3%	11.4 ± 0.5	+6.6%
**Wr. depth (μm)**	**O**	349.8 ± 14.1	350.4 ± 14.4	+0.1%	325.0 ± 13.4 ***	−6.9% ***	345.7 ± 16.7	347.8 ± 16.7	+0.8%	349.1 ± 16.5	+1.4%
	**A**	291.0 ± 9.5	289.3 ± 9.9	−0.6%	269.9 ± 10.3 ***	−7.5% ***	277.3 ± 12.8	282.7 ± 13.3 *	+2.0%	281.1 ± 12.4	+1.8%
	**C**	431.6 ± 21.5	435.4 ± 21.4	+1.1%	401.6 ± 19.5 **	−6.2% **	440.9 ± 24.7	438.4 ± 25.4	−0.8%	443.7 ± 24.5	+0.8%

Moist, Skin moisturization; Rad, Skin radiance; ITA°, Individual Typology Angle; Wr. depth, wrinkle depth.

**Table 3:** Skin elasticity results. The intragroup (vs. baseline) statistical analysis is reported near the raw data, while the intergroup (active vs. placebo) statistical analysis is reported near the % variation (Δ%). The statistical analysis is reported as follows: * *p* < 0.05, ** *p* < 0.01, and *** *p* < 0.005. Legend. D0, baseline; D14, follow-up visit after 15 days of product use; D57, follow-up visit after 56 days of product use. O, Overall (Asian and Caucasian subjects); A, Asian; C, Caucasian.

		Active					Placebo				
		D0	D15	Δ%	D57	Δ%	D0	D15	Δ%	D57	Δ%
**R0 (mm)**	**O**	0.321 ± 0.013	0.294 ± 0.012 ***	−8.2% ***	0.269 ± 0.011 ***	−15.6%	0.322 ± 0.013	0.327 ± 0.013 *	+2.0%	0.330 ± 0.014	+3.1%
	**A**	0.257 ± 0.006	0.254 ± 0.006 ***	−9.3% ***	0.215 ± 0.007 ***	−15.2% ***	0.251 ± 0.007	0.257 ± 0.008	+2.5%	0.260 ± 0.010	+3.8%
	**C**	0.413 ± 0.017	0.384 ± 0.014 **	−6.5% ***	0.343 ± 0.012 ***	−16.0% ***	0.420 ± 0.012	0.425 ± 0.013	+1.2%	0.429 ± 0.014	+3.8%
**R2 (%)**	**O**	0.593 ± 0.015	0.626 ± 0.016 ***	+5.9% ***	0.671 ± 0.015 ***	+13.9% ***	0.582 ± 0.013	0.583 ± 0.013	+0.1%	0.577 ± 0.014	−0.9%
	**A**	0.520 ± 0.010	0.551 ± 0.010 ***	+6.2% ***	0.598 ± 0.011 ***	+15.5% ***	0.522 ± 0.010	0.523 ± 0.011	0.0%	0.518 ± 0.014	−0.9%
	**C**	0.695 ± 0.017	0.730 ± 0.018 **	+5.5% **	0.773 ± 0.018 ***	+11.6% ***	0.665 ± 0.014	0.667 ± 0.014	+0.2%	0.0659 ± 0.015	−0.8%
**R5 (%)**	**O**	0.449 ± 0.016	0.514 ± 0.018 ***	+16.8% ***	0.587 ± 0.022 ***	+33.4% ***	0.447 ± 0.016	0.438 ± 0.017	−2.6%	0.441 ± 0.016	−1.2%
	**A**	0.504 ± 0.012	0.579 ± 0.016 ***	+15.9% ***	0.668 ± 0.019 ***	+34.3% ***	0.500 ± 0.011	0.485 ± 0.015	−3.1%	0.491 ± 0.015	−1.8%
	**C**	0.372 ± 0.025	0.424 ± 0.025 **	+17.1% ***	0.475 ± 0.029 ***	+31.0% ***	0.375 ± 0.024	0.372 ± 0.026	−1.3%	0.372 ± 0.024	−0.6%
**R9 (mm)**	**O**	0.040 ± 0.001	0.035 ± 0.001 ***	−12.1% ***	0.030 ± 0.001 ***	−25.4% ***	0.039 ± 0.001	0.041 ± 0.002	6.5%	0.041 ± 0.002	+5.9%
	**A**	0.039 ± 0.002	0.034 ± 0.002 ***	−12.1% ***	0.029 ± 0.002 ***	−25.8% ***	0.039 ± 0.001	0.042 ± 0.002 *	+10.3%	0.041 ± 0.002	+8.2%
	**C**	0.041 ± 0.002	0.037 ± 0.003 **	−11.9% *	0.031 ± 0.002 ***	−23.8% ***	0.039 ± 0.002	0.039 ± 0.002	+10.3%	0.041 ± 0.003	+8.2%

## Data Availability

The data presented in this study are available upon request from the corresponding author. The data are not publicly available since they are property of the sponsor of the study (Bionap Srl, 95032 Piano Tavola Belpasso CT, Italy).

## Supplementary Materials

The following supporting information can be downloaded at: https://www.mdpi.com/article/10.3390/nu14112241/s1. Table S1: Inclusion and exclusion criteria; Figure S1: Measurements’ sites; Figure S2: Solar simulator spectral characteristics; Figure S3: Skin elasticity parameters; Table S2: Subjects’ baseline demographic and clinical characteristics; Table S3: UVA-stimulated MDA results; Table S4: Wrinkle length, area, and wrinkle count; Table S5: Anthropometric parameters.
