# Supplementary material for: Photoprotective and Antiaging Effects of a Standardized Red Orange (Citrus sinensis (L.) Osbeck) Extract in Asian and Caucasian Subjects: A Randomized, Double-Blind, Controlled Study
Source: Nutrients. 2022 May 27;14(11):2241. doi: 10.3390/nu14112241 (PMC9182634; doi:10.3390/nu14112241)
Supplement: Supplementary file 1 [file nutrients-14-02241-s001.zip › nutrients-1725625-supplementary.pdf]

# SUPPLEMENTARY INFORMATION

**Photoprotective and Antiaging Effects of a Standardized Red Orange (Citrus sinensis (L.) Osbeck) Extract in Asian and Caucasian Subjects: A Randomized, Double-Blind, Controlled Study.**

Vincenzo Nobile*, Andrea Burioli, Sara Yu, Shi Zhifeng, Enza Cestone, Violetta Insolia, Vincenzo Zaccaria* and Giuseppe Antonio Malfa.

**Supplementary Table S1.** Inclusion and exclusion criteria.

| No. | Inclusion criteria |
|---|---|
| 01 | Female and male healthy subjects (between 30-50% male and 50-70% female) |
| 02 | 35 ≤ age ≤ 65 years old |
| 03 | 40% Caucasian (skin phototype* I to III) and 60% Asian (skin phototype* III to V) ethnicity |
| 04 | Mild to moderate skin ageing sign including dark spots (grade 2-4; Skin Aging Atlas by Bazin R.) |
| 05 | Registration with health social security or health social insurance (if required by national regulations) |
| 06 | Promise to not use during all the study period topic products/food supplements with similar effect to that one of the product to be tested (antioxidant) |
| 07 | Commitment to sign the informed consent form (ICF) |
| 08 | Truthfulness of the personal information declared to the Investigator |
| 09 | Ability to understand the language used in the investigation centre and the information given |
| 10 | No recent participation in any other similar study |
| 11 | No sun exposure (both natural and artificial) for at least two months before study start, |
| 12 | Absence of sunburn, suntan, scars or other active dermal lesions on the area selected for test purposes |
| 13 | Color uniformity of the test area (without nevi, blemishes or solar lentigo and without hair) |
| 14 | Promise to not change the normal daily routine, effective contraceptive therapy |
| 15 | Ability to comply with the protocol and follow protocol's constraints and specific requirements |
| **No.** | **Exclusion criteria** |
| 01 | Breastfeeding, pregnancy (or unwillingness to take necessary precautions to avoid pregnancy during the study and for at least 3 months before the inclusion visit [for women of childbearing potential] |
| 02 | Staring or changing oestrogen-progesterone contraception or hormonal treatment, within the 3 months prior to the study or foreseeing it for the duration of the study |
| 03 | Allergies or sensitivity to cosmetic products, toiletries, sunscreens, and/or topical drugs |
| 04 | Dermatological problems in the test area |
| 05 | Pharmacological treatments (both locally or systemically) |
| 06 | Positive anamnesis for atopy (allergic hypersensitivity affecting parts of the body not in direct contact with the allergen) |
| 07 | Use of self-tanning products for at least one month before study start |
| 08 | Habit to use tanning beds, |
| 09 | Medication with photosensitizing potential, drugs, and/or food supplements able to induce skin coloring, corticoids, currently or during the month before the study start |
| 10 | Participation in another clinical trial within the last two weeks prior to the inclusion visit and taking part or planning to participate to another clinical trial during the study in the same or another investigation center |
| 11 | Deprivation of freedom by administrative or legal decision or under guardianship |
| 12 | Unavailability to be contacted in case of emergency |
| 13 | Admission in a sanitary or social facility |
| 14 | Planning a hospitalization during the study |
| 15 | Impaired immune system due to immunosuppressive diseases, or use of immunosuppressive medication |
| 16 | Acute, chronic or progressive illness liable to interfere with the study data or considered by the Investigator hazardous for the subject or incompatible with the study requirements |
| 17 | History of severe reactions from exposure to sunlight (i.e., polymorphous light eruption) |
| 18 | Long-treatment or intending to have one, in particular with aspirin, products containing aspirin, corticoids, beta blockers (including eye drops), immuno-suppressive and/or desensitization drugs or under any treatment considered by the Investigator liable to interfere with the study data or incompatible with the study requirements |
| 19 | Vaccination within the 3 weeks prior to the study or intending to be vaccinated during the study |
| 20 | Any surgery, chemical or physical treatment on the experimental area within the 12 months prior to the study or foreseeing it for the duration of the study |
| 21 | Personal history of atopic dermatitis, urticaria or severe skin reaction to cosmetics, drugs or domestic products or confirmed contact dermatitis or food allergy |
| 22 | Artificial UV exposure or excessive exposure to natural sunlight or within the 2 weeks prior to the study or foreseeing UV exposures for the duration of the study (at Investigator's judgment) |

* The skin phototype is a constitutional characteristic and is related to constitutional color (depending on the amount of melanin pigment in the skin) and the effect of exposure to ultraviolet radiation (tanning). According Fitzpatrick the skin phototype can be classified on a scale from 1 to 6, as follows: I subject with pale white skin, blue/hazel eyes, blond/red hair (always burns and does not tan); II subjects with fair skin, blue eyes (burns easily, tans poorly); III subjects with darker white skin (tans after initial burn); IV subjects with light brown skin (burns minimally, tans easily); V subjects with brown skin (rarely burns, tans darkly easily); VI dark brown or black skin (never burns, always tans darkly.

| Anatomic area | Measured parameter | Notes |
|---|---|---|
| Face<br>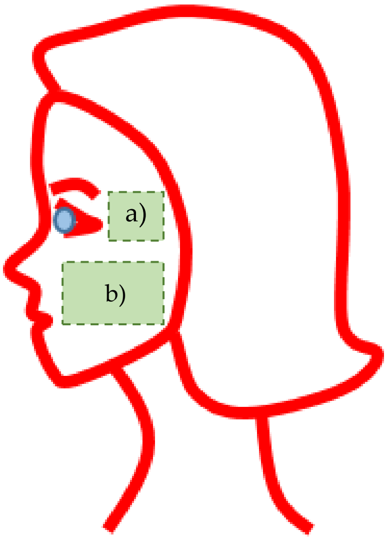 | ▪ The following parameters area measured in the "b" test area<br>▪ Skin elasticity<br>▪ Transepidermal water loss<br>▪ Skin radiance<br>▪ Skin moisturization<br>▪ Intensity of melanin staining*<br><br>The following parameters area measured in the "a" test area<br>▪ Wrinkle depth, length, area, and wrinkle count<br>▪ Intensity of melanin staining* | * Intensity of melanin staining is measured inside the dark spot.<br>a) "*crow's feet*" area<br>b) cheek |
| Back<br>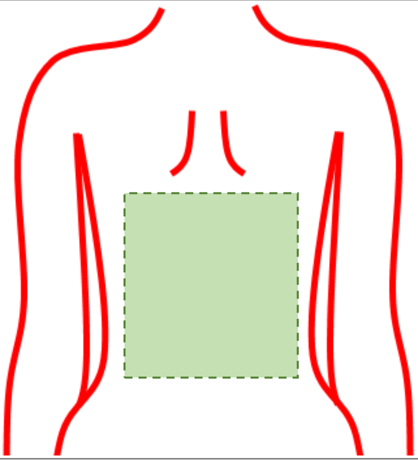 | ▪ Minimal Erythema Dose<br>▪ UV-induced erythema | 1. Up to six subsites (yellow rectangle with circles inside) can be randomized over the test area. One subsite is exposed to 6 UV doses in order to determine the MED.<br>2. One subsite is exposed to 1 MED UV dose in 3 small areas (circles inside the rectangle) in order to measure the UV-induced erythema. |
| Legs<br>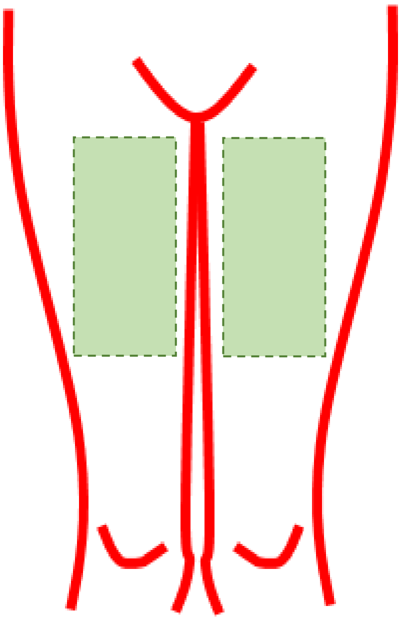 | ▪ Skin stripping<br>▪ UVA exposure<br>▪ Total skin antioxidant capacity<br>▪ Lipoperoxides | Skin stripping are taken in a subsite (yellow rectangle) inside test area. The same is for UVA exposure<br>Shaving (if needed) for male subjects. |

**Supplementary Figure S1.** Measurement's site.

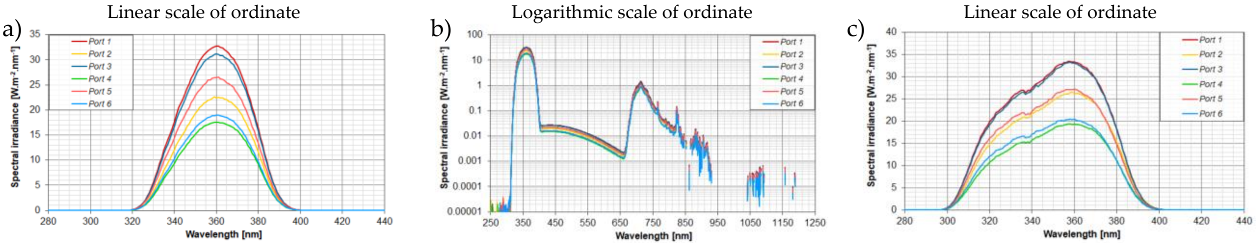


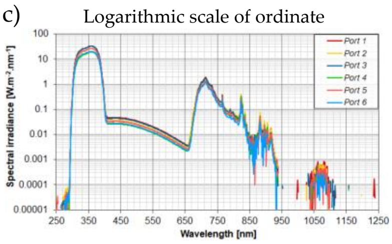


d)

| Parameter | Limits | | Measurement results | | | | | |
|---|---|---|---|---|---|---|---|---|
| | Lower | Upper | Port 1 | Port 2 | Port 3 | Port 4 | Port 5 | Port 6 |
| UVB / UV | - | 0,1 % | 0.02% | 0.02% | 0.02% | 0.02% | 0.02% | 0.02% |
| **UVA2 / UVA** | **8%** | **20%** | **10.4%** | **10.1%** | **10.3%** | **10.1%** | **10.5%** | **10.3%** |
| UVA1 / UVA | 80% | 92% | 89.6% | 89.9% | 89.7% | 89.9% | 89.5% | 89.7% |
| $E_{400nm \leq \lambda, \leq 1600nm} / E_{total}$ | - | 5% | 4.2% | 4.4% | 4.1% | 4.7% | 4.2% | 4.4% |
| Total irradiance [W·m$^{-2}$] | - | 1600 | 1380 | 959 | 1316 | 753 | 1122 | 809 |
| **In-vivo UVA ISO 24442** | | | **Pass** | **Pass** | **Pass** | **Pass** | **Pass** | **Pass** |

e)

| Parameter | Limits | | Measurement results | | | | | |
|---|---|---|---|---|---|---|---|---|
| | Lower | Upper | Port 1 | Port 2 | Port 3 | Port 4 | Port 5 | Port 6 |
| RCEE% (< 290 nm) | - | 0.1% | 0.0% | 0.0% | 0.0% | 0.0% | 0.0% | 0.0% |
| RCEE% (290 - 300 nm) | 1% | 8% | 5.2% | 4.8% | 5.2% | 5.5% | 5.4% | 5.5% |
| RCEE% (290 - 310 nm) | 49% | 65% | 56.7% | 55.0% | 56.6% | 56.5% | 57.4% | 57.1% |
| **RCEE% (290 - 320 nm)** | **85%** | **90%** | **86.9%** | **85.8%** | **86.8%** | **86.2%** | **87.1%** | **86.9%** |
| RCEE% (290 - 330 nm) | 91.5% | 95.5% | 92.6% | 91.9% | 92.5% | 92.1% | 92.8% | 92.6% |
| RCEE% (290 - 340 nm) | 94% | 97% | 95.0% | 94.4% | 94.9% | 94.5% | 95.1% | 94.9% |
| RCEE% (290 – 400 nm) | 99.9% | 100% | 100.0% | 100.0% | 100.0% | 100.0% | 100.0% | 100.0% |
| Ratio UVA2 / UV | 20% | - | 24.6% | 24.0% | 24.5% | 23.9% | 24.6% | 24.6% |
| Ratio UVA1 / UV | 60% | - | 65.7% | 67.0% | 65.9% | 67.2% | 65.6% | 65.7% |
| Total irradiance ISO [W·m$^{-2}$] | - | 1600 | 2 079 | 1 647 | 2 040 | 1 207 | 1 680 | 1 272 |
| Total irradiance FDA [W·m$^{-2}$] | - | 1500 | | | | | | |
| **ISO 24444** | | | **Pass** | **Pass** | **Pass** | **Pass** | **Pass** | **Pass** |
| **FDA (2011)** | | | **Pass** | **Pass** | **Pass** | **Pass** | **Pass** | **Pass** |

**Supplementary Figure S2.** Solar simulator spectral characteristics. a) Spectral irradiance, filter setting "UVA". b) Spectral irradiance, filter setting "UVA". c) Spectral irradiance, filter setting "UVA+B". d) Spectral irradiance, filter setting "UVA+B". e) Compliance of the device under test EN ISO 24442 specifications. f) Compliance of the device under test with the specifications of ISO 24444 and FDA 2011 *in vivo* SPF test methods.

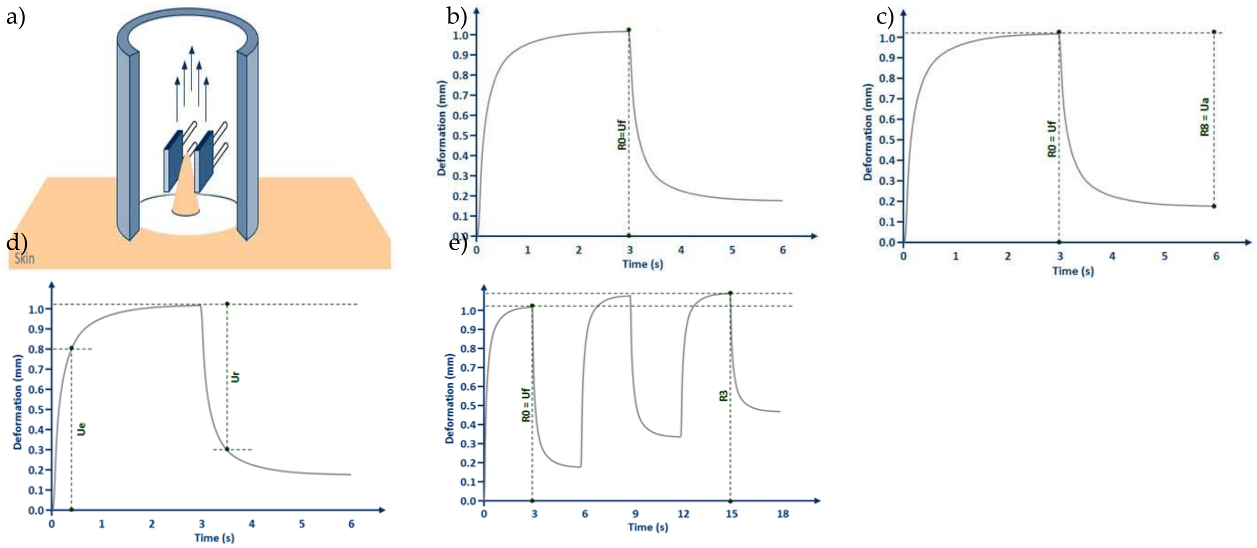


**Supplementary Figure S3.** Skin elasticity parameters. a) Skin elasticity measurement process. b) R0 (skin distensibility) represents the passive behavior of the skin to force (i.e., gravity). Conceptually R0 parameter is correlated to skin firmness. c) R2 (Ua/Uf, gross elasticity or overall elasticity) represents the ability of the skin to return to its basal state. d) R5 (Ur/Ue, net elasticity) represent the elastic recovery of the skin to its basal state due to its elastic component after deformation. The parameter decays with ageing and it is independent of the skin thickness. e) R9 (R3 - R0, skin tiring effect) represent the increase of deformation of the skin after repetitive stimulation and it is related to skin tiring effects.

**Supplementary Table S2.** Subjects' baseline demographic and clinical characteristics. O Overall (Asian and Caucasian subjects); A Asian; C Caucasian.

| | Active | | | Placebo | | | |
|---|---|---|---|---|---|---|---|
| | **O** | **A** | **C** | **O** | **A** | **C** | **Units** |
| **Sex** | | | | | | | |
| **Male, % (n)** | 40.0 (22) | 40.6 (13) | 39.1 (9) | 40.0 (22) | 40.6 (13) | 39.1 (9) | % (no.) |
| **Female, % (n)** | 60.0 (33) | 59.4 (19) | 60.9 (14) | 60.0 (33) | 59.4 (19) | 60.9 (14) | % (no.) |
| **Age** | 53.4±1.1 | 52.3±1.6 | 55.1±1.4 | 53.5±1.2 | 52.7±1.7 | 54.5±1.7 | Years |
| **MED** | 33.7±2.0 | 31.8±2.7 | 36.4±2.9 | 34.8±1.9 | 38.3±3.3 | 32.3±2.1 | mJ/cm$^2$ |
| **Skin moisturization** | 53.7±2.2 | 61.7±2.1 | 42.6±3.3 | 55.1±2.2 | 62.6±2.3 | 44.7±3.0 | cu |
| **Skin elasticity** | | | | | | | |
| **R0** | 0.321±0.013 | 0.257±0.006 | 0.413±0.017 | 0.322±0.013 | 0.251±0.007 | 0.420±0.012 | mm |
| **R2** | 0.593±0.015 | 0.520±0.010 | 0.695±0.017 | 0.582±0.013 | 0.522±0.010 | 0.665±0.014 | % |
| **R5** | 0.449±0.016 | 0.504±0.012 | 0.372±0.025 | 0.447±0.016 | 0.500±0.011 | 0.375±0.024 | % |
| **R9** | 0.040±0.001 | 0.039±0.002 | 0.041±0.002 | 0.039±0.001 | 0.039±0.001 | 0.039±0.002 | mm |
| **Skin radiance** | 10.9±0.5 | 10.5±0.7 | 11.4±0.9 | 11.6±0.5 | 10.6±0.6 | 13.0±0.9 | au |
| **ITA° (dark spots)** | 23.0±1.3 | 23.4±1.8 | 22.4±1.7 | 23.5±1.1 | 24.1±1.7 | 22.8±1.4 | ° |
| **TEWL** | 12.7±0.5 | 13.3±0.7 | 11.9±0.6 | 12.2±0.5 | 13.2±0.7 | 10.9±0.5 | g/h/m$^2$ |
| **FRAP** | 329.0±8.4 | 321.2±11.3 | 339.9±12.6 | 333.8±9.3 | 331.3±10.5 | 337.2±17.0 | mm $Fe^{2+}$ |
| **MDA** | 16.8±0.6 | 16.4±0.8 | 17.5±0.9 | 16.9±0.6 | 17.3±0.8 | 16.4±0.8 | µM (MDA) |
| **Wrinkles** | | | | | | | |
| **Depth** | 349.8±14.1 | 291.0±9.5 | 431.6±21.5 | 345.7±16.7 | 277.3±12.8 | 440.9±24.7 | µm |
| **Count** | 6.9±0.3 | 5.9±0.3 | 8.2±0.4 | 6.7±0.3 | 5.7±0.3 | 8.1±0.4 | no. |
| **Area** | 38.39±1.74 | 31.69±1.30 | 47.72±2.77 | 36.79±1.71 | 30.08±1.13 | 46.12±2.78 | mm$^2$ |
| **Length** | 64.4±2.5 | 54.6±2.3 | 78.11±3.3 | 64.4±2.2 | 56.4±2.2 | 75.6±3.1 | mm |
| **Weight** | 64.3±1.4 | 63.1±1.8 | 66.0±2.1 | 65.8±1.4 | 63.8±1.6 | 68.4±2.4 | Kg |
| **Height** | 1.62±0.01 | 1.61±0.02 | 1.63±0.02 | 1.63±0.01 | 1.61±0.01 | 1.66±0.02 | m |
| **BMI** | 24.5±0.3 | 24.4±0.4 | 24.7±0.5 | 24.6±0.4 | 24.5±0.4 | 24.7±0.6 | |
| **Circumferences** | | | | | | | |
| **Waist** | 85.7±1.5 | 85.1±1.6 | 86.4±2.7 | 85.7±1.5 | 83.7±1.4 | 88.5±3.1 | cm |
| **Hip** | 97.1±1.3 | 92.4±0.9 | 103.7±2.1 | 97.8±0.9 | 94.3±0.7 | 102.7±1.4 | cm |

Data are average (± standard error) or number of subjects (n or %). Legend. MED Minimal Erythema Dose; ITA° Individual Typology Angle; FRAP Ferric Reducing Antioxidant Parameter; MDA Malonyldialdehyde; BMI Body Mass Index; cu corneometric units; au arbitrary units.

**Supplementary Table S3.** UVA-stimulated MDA results. The intergroup (active vs. placebo) statistical analysis is reported near the % variation. The statistical analysis is reported as follows: * p<0.05, ** p<0.01, and *** p<0.001. Legend, D-1+4h is the day before the basal visit (4 hours after the UV exposure), D0 is the day after D-1 (24 hours after UV exposure), D14+4h is 4 hours after UV exposure at D14, D15 follow-up visit after 14 days of product use (and 24 hours after UV exposure at D14), D56+4h is 4 hours after UV exposure at D56, D57 follow-up visit after 56 days of product use (and 24 hours after UV exposure at D56). O Overall (Asian and Caucasian subjects); A Asian; C Caucasian.

| | Active | | | | | | Placebo | | |
|---|---|---|---|---|---|---|---|---|---|
| | **O** | | **A** | | **C** | | **O** | **A** | **C** |
| **D-1+4h** | +56.0% | | +58.2% | | +53.0% | | +53.3% | +55.0% | +50.9% |
| **D0** | +28.5% | | +29.3% | | +27.2% | | +28.9% | +28.5% | +29.3% |
| **D14+4h** | +53.5% | | +57.2% | | +48.4% | | +50.6% | +51.4% | +49.6% |
| **D15** | +22.4% | | +22.9% | | +21.7% | | +26.6% | +29.2% | +23.0% |
| **D56+4h** | +40.7% | ** | +41.1% | ** | +40.1% | | +51.8% | +51.6% | +52.2% |
| **D57** | +14.5% | *** | +14.7% | *** | +14.1% | * | +26.7% | +27.7% | +25.2% |

**Supplementary Table S4.** Wrinkle length, area, and wrinkle count. Legend D0 baseline, D14 follow-up visit after 15 days of product use, D57 follow-up visit after 56 days of product use. O Overall (Asian and Caucasian subjects); A Asian; C Caucasian. Δ% percentage variation vs. baseline.

| | | Active | | | | | Placebo | | | | |
|---|---|---|---|---|---|---|---|---|---|---|---|
| | | **D0** | **D15** | **Δ%** | **D57** | **Δ%** | **D0** | **D15** | **Δ%** | **D57** | **Δ%** |
| **Length (mm)** | **O** | 64.4±2.5 | 64.4±2.4 | +0.3% | 63.6±2.3 | -0.8% | 64.4±2.2 | 64.8±2.2 | +0.7% | 65.0±2.2 | +1.2% |
| | **A** | 54.6±2.3 | 54.7±2.2 | +0.5% | 54.2±2.2 | -0.2% | 56.4±2.2 | 57.1±2.3 | +1.3% | 57.2±2.2 | +1.6% |
| | **C** | 78.11±3.3 | 77.8±3.2 | -0.2% | 76.6±3.0 | -1.4% | 75.6±3.1 | 75.6±3.1 | +0.1% | 76.0±3.0 | +0.8% |
| **Area ($mm^2$)** | **O** | 38.39±1.74 | 38.43±1.71 | +0.5% | 37.50±1.60 | -1.5% | 36.79±1.71 | 37.04±1.68 | +1.1% | 37.15±1.69 | +1.3% |
| | **A** | 31.69±1.30 | 31.76±1.25 | +0.7% | 31.13±1.30 | -1.4% | 30.08±1.13 | 30.55±1.16 | +1.8% | 30.46±1.16 | +1.4% |
| | **C** | 47.72±2.77 | 47.71±2.73 | +0.2% | 46.37±2.37 | -1.6% | 46.12±2.78 | 46.07±2.73 | 0.0% | 46.46±2.70 | +1.2% |
| **Count (no.)** | **O** | 6.9±0.3 | 6.8±0.3 | -0.1% | 6.8±0.3 | +0.1% | 6.7±0.3 | 6.7±0.3 | -0.1% | 6.7±0.3 | +0.2% |
| | **A** | 5.9±0.3 | 5.9±0.3 | +0.3% | 5.9±0.3 | +0.3% | 5.7±0.3 | 5.8±0.3 | +0.4% | 5.7±0.2 | +0.7% |
| | **C** | 8.2±0.4 | 8.0±0.4 | -1.4% | 8.1±0.4 | +0.2% | 8.1±0.4 | 8.0±0.4 | -0.3% | 8.1±0.4 | -0.3% |

**Supplementary Table S5.** Anthropometric parameters. Legend D0 baseline, D14 follow-up visit after 15 days of product use, D57 follow-up visit after 56 days of product use. O Overall (Asian and Caucasian subjects); A Asian; C Caucasian. Inside the bracket is reported the intragroup (vs. baseline) statistical analysis.

| | | Weight (Kg) | | | Height (m) | | | BMI | | |
|---|---|---|---|---|---|---|---|---|---|---|
| | | **D0** | **D15** | **D57** | **D0** | **D15** | **D57** | **D0** | **D15** | **D57** |
| **O** | **Active (n=55)** | 64.3±1.4 | 64.5±1.4 [p=0.3164] | 64.7±1.4 [p=0.4871] | 1.62±0.01 | 1.62±0.01 [p=1.0000] | 1.62±0.01 [p=1.0000] | 24.5±0.3 | 24.6±0.3 [p=0.2646] | 24.6±0.3 [p=0.5214] |
| | **Placebo (n=55)** | 65.8±1.4 | 65.7±1.4 | 66.0±1.4 | 1.63±0.01 | 1.63±0.01 | 1.63±0.01 | 24.6±0.4 | 24.6±0.4 | 24.7±0.4 |
| **A** | **Active (n=32)** | 63.1±1.8 | 63.3±1.8 [p=0.1351] | 63.6±1.8 [p=0.6829] | 1.61±0.02 | 1.61±0.02 [p=1.0000] | 1.61±0.02 [p=1.0000] | 24.4±0.4 | 24.5±0.4 [p=0.1230] | 24.6±0.4 [p=.7005] |
| | **Placebo (n=32)** | 63.8±1.6 | 63.8±1.6 | 64.2±1.6 | 1.61±0.01 | 1.61±0.01 | 1.61±0.01 | 24.5±0.4 | 24.5±0.4 | 24.7±0.4 |
| **C** | **Active (n=32)** | 66.0±2.1 | 66.1±2.1 [p=0.7173] | 64.7±1.4 [p=0.4441] | 1.63±0.01 | 1.63±0.01 [p=1.0000] | 1.63±0.01 [p=1.0000] | 24.7±0.5 | 24.7±0.5 [p=0.2899] | 24.7±0.6 [p=0.2668] |
| | **Placebo (n=32)** | 68.4±2.4 | 68.5±2.4 | 68.5±2.4 | 1.66±0.02 | 1.66±0.02 | 1.66±0.02 | 24.7±0.6 | 24.7±0.6 | 24.7±0.6 |

| | | Waistline circumference (cm) | | | Hip circumference (cm) | | |
|---|---|---|---|---|---|---|---|
| | | D0 | D15 | D57 | D0 | D15 | D57 |
| **O** | **Active (n=55)** | 85.7±1.5 | 85.5±1.5 [p=0.8662] | 85.6±1.4 [p=0.5753] | 97.1±1.3 | 97.2±1.3 [p=0.7171] | 97.3±1.3 [p=0.8372] |
| | **Placebo (n=55)** | 85.7±1.5 | 85.5±1.5 | 85.5±1.5 | 97.8±0.9 | 97.9±0.9 | 97.9±0.9 |
| **A** | **Active (n=23)** | 85.1±1.6 | 84.8±1.6 [p=0.3479] | 85.0±1.6 [p=0.8566] | 92.4±0.9 | 92.5±0.9 [p=0.4295] | 92.6±1.0 [p=0.3172] |
| | **Placebo (n=23)** | 83.7±1.4 | 83.7±1.4 | 83.7±1.4 | 94.3±0.7 | 94.2±0.7 | 94.2±0.8 |
| **C** | **Active (n=32)** | 86.4±2.7 | 86.4±2.7 [p=0.2899] | 86.4±2.7 [p=0.2668] | 103.7±2.1 | 103.8±2.1 [p=0.5297] | 103.7±2.1 [p=0.1921] |
| | **Placebo (n=32)** | 88.5±3.1 | 88.0±2.9 | 88.0±2.9 | 102.7±2.9 | 103.0±1.5 | 103.1±1.4 |
